# Operating Comfort Prediction Model of Human-Machine Interface Layout for Cabin Based on GEP

**DOI:** 10.1155/2015/896072

**Published:** 2015-09-10

**Authors:** Li Deng, Guohua Wang, Bo Chen

**Affiliations:** ^1^School of Mechatronic Engineering, Southwest Petroleum University, Chengdu 610500, China; ^2^State Key Laboratory of Oil and Gas Reservoir Geology and Exploitation, Southwest Petroleum University, Chengdu 610500, China

## Abstract

In view of the evaluation and decision-making problem of human-machine interface layout design for cabin, the operating comfort prediction model is proposed based on GEP (Gene Expression Programming), using operating comfort to evaluate layout scheme. Through joint angles to describe operating posture of upper limb, the joint angles are taken as independent variables to establish the comfort model of operating posture. Factor analysis is adopted to decrease the variable dimension; the model's input variables are reduced from 16 joint angles to 4 comfort impact factors, and the output variable is operating comfort score. The Chinese virtual human body model is built by CATIA software, which will be used to simulate and evaluate the operators' operating comfort. With 22 groups of evaluation data as training sample and validation sample, GEP algorithm is used to obtain the best fitting function between the joint angles and the operating comfort; then, operating comfort can be predicted quantitatively. The operating comfort prediction result of human-machine interface layout of driller control room shows that operating comfort prediction model based on GEP is fast and efficient, it has good prediction effect, and it can improve the design efficiency.

## 1. Introduction

As a complicated human-machine environment system, cabin is the working space to perform the task of observation and manipulation, centralizing display instrument, manipulator, signal, alarm, and other terminal interfaces, for example, aerospace manned cabin, deep-sea submersibles cabin, engineering machinery cab, drilling rig control room, control room in nuclear power plant, and automobile cabin. The internal structure of these cabins is complex; the operators need to rely on vision, hearing, and touching to get information from the instruments and the outside world and make judgments quickly then immediately through locomotive organ such as hand and foot to manipulate correctly. In this state, the comfort of operating posture is an important factor to affect the operators' work load, fatigue, health, and even safety, which should be considered in the human-machine interface design with emphasis. In the human-machine interface design for cabin, the layout design of all sorts of manipulators directly determines different operating postures, and the different operating postures directly affect operating comfort, convenience, and efficiency. Comfortable operating posture is advantageous to keep good matching relation between locomotive organ and manipulators. Therefore, the operating comfort is an important basis to evaluate layout design of human-machine interface for cabin [1].

Comfortable feeling is a kind of subjective feeling combined with the experience between physiological and psychological perception and affected by various factors such as work environment, duration, and different task [2–5]. The comfort evaluation research is usually divided into two categories: subjective evaluation and objective evaluation. Through a variety of evaluation scale directly, subjective evaluation has the advantages of low cost and simple operation steps. However, relying on subjective description of different subjects, the feeling of subjects themselves, the difference of patience, the mood, and other subjective factors and test environment have influence on the comfort evaluation results obtained by statistical analysis; the evaluation results given by different subjects may have difference [6–11]. Objective evaluation can be proceeded by physical model experiment using ergonomic experiment equipment such as 3D motion capture system and pressure distribution measurement system and through anthropometry, biomechanical characteristic, body pressure distribution, operating posture and so on; comfortable feeling can be obtained based on observation, recording, and measuring objectively and indirectly [12–17].

At present, operating posture research focuses on using camera, driving posture monitoring system, 3D motion analysis, and other equipment to conduct the measurement, statistics, and analysis of postures. This kind of method is time-consuming and expensive and cannot be used in the design process early. Moreover, there is lack of effective modeling method in the comfort evaluation of operating posture. In order to evaluate the layout design scheme of human-machine interface for cabin in the early stage of design process and reduce the times of rework and production of physical prototype, shorten the cycle from design to manufacturing and cost [18]; on the basis of Chinese adult's body parameters to establish virtual human body model, this paper attempts to use CATIA software to do simulation experiment [19] and simulate and analyze the human operating postures. Then, the relation model of joint angles and operating comfort is established based on GEP algorithm. Through the limb angles to predict the comfort of operating posture, a simple and effective evaluation method is provided to evaluate the human-machine interface layout.

## 2. Prediction Method of Operating Comfort Based on GEP

### 2.1. Outline of the Proposed Method

**Figure 1:**
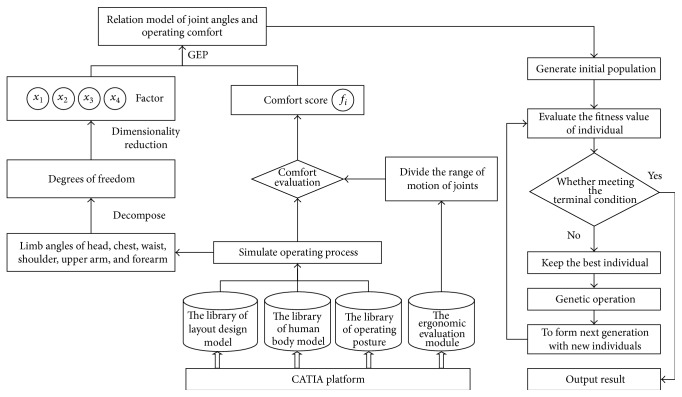
Prediction method of operating comfort of human-machine interface layout for cabin based on GEP.

The implementation steps of the proposed method are shown in Figure 1. First of all, take CATIA software as the simulation platform, build the library of layout design model, human body model, and operating posture, simulate the operating process, output the limb angles of head, chest, waist, shoulder, upper arm, and forearm in various operating postures, and use the ergonomic evaluation module of CATIA to evaluate the comfort. Second, analyze the data collected from simulation, factor analysis is used to reduce the dimension of variables affecting the operating comfort, effectively reduce the input variables of prediction model, and eliminate the correlation between the input data. Then, using the function finding ability of GEP algorithm, establish the relation model of joint angles and operating comfort. Finally, the example of the operating comfort prediction of driller in driller control room verifies the effectiveness of the proposed method.

### 2.2. Evaluation Principle of Operating Comfort

#### 2.2.1. Operating Posture

**Figure 2:**
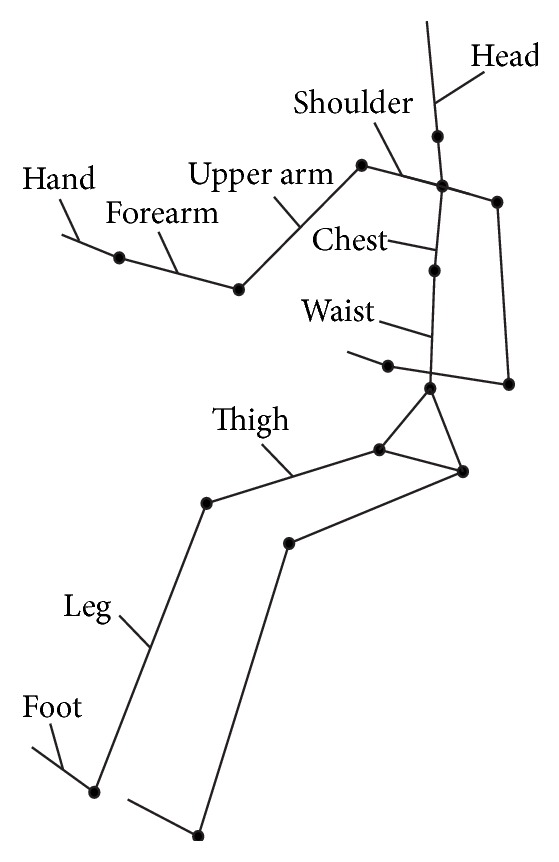
The simplified model of human skeletal system.

Through limb angles to describe the postures, joint angles are taken as independent variables to establish a comfort model of operating posture. Using mathematical method to describe each part size of the human body and relative position, it is used for the analysis of working posture and operating range and has nothing to do with the characteristics of the human body volume [20]. Joint connects different limb and is the pivot to transmit force and torque, allowing the body to move normally. Taking the joints as points and the bones between the joints as chains, the human torso and limbs are connected, and the skeletal system of human body modeling is formed. As shown in Figure 2, the human body skeleton model is simplified as follows: the upper limb is divided into upper arm, forearm, and hand; lower limb is divided into thigh, leg, and feet; trunk is divided into head, shoulder, chest, and waist.

#### 2.2.2. The Comfort of Joint

The human body's different joint angles form different postures. To study comfortable working posture, the comfortable joint angles of the human body should be studied firstly. Most operation in cabin is given priority to sitting posture, so this paper chooses six parameters which are the most close to operating comfort of upper limb as variables of ergonomic characteristic. The six parameters are head, chest, waist, shoulder, upper arm, and forearm. The joint angles between adjacent limbs are used to describe the working posture.

**Figure 3:**
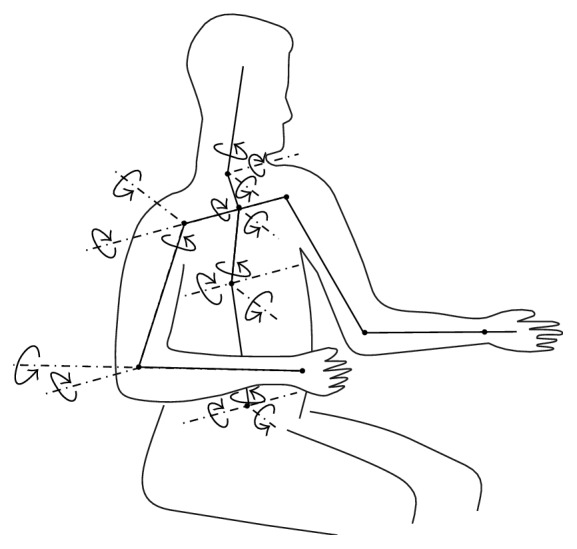
Degrees of freedom of joints.

The human body has many joints, and every joint has multiple degrees of freedom (DOF); thus many motions can be realized precisely. Joint motion can be regarded as the rotation around axis; the type of joints determines its form of motion. According to the DOF of the activity of articular surface relative to the joints of each other, the joints are divided into three kinds: DOF 1, DOF 2, and DOF 3. The DOF of joints involved in upper limb operating is shown in Figure 3.

Range of motion (ROM) of joints depends on statistical numerical [21]; it can be expressed as *R*_*i*_^*L*^ ≤ *R*_*i*_ ≤ *R*_*i*_^*U*^, *i* = 1, 2,…, *n*, where *L* and *U* are the lower limit and upper limit of ROM of joints, respectively, and *n* is the number of joints. Discomfort is the value function where the respective deviation from the center position of a joint determines the discomfort. The comfort of center position of joint is expressed as *R* ^*C*^; then the discomfort relative to comfortable position can be represented as *R* − *R* ^*C*^. The comfortable feeling of each joint is not the same; weight *W*_*i*_ should be introduced to express the relative comfortable degree between each joint; weighted value is taken as judgment function of comfort:

(1) $$\begin{array}{c} f\left(\;R\;\right)=\overset{n}{\underset{i=1}{\sum }}W_{i}\left|\;R_{i}-R_{i}^{C}\;\right|, \end{array}$$

where ∑_*i*=1_^*n*^ *W*_*i*_ = 1.

Driving posture is an important issue in vehicle design process; many scholars and institutions have carried out a number of studies on optimal driving posture, preferred angles, seat comfort, and so on. Referring to the research results in vehicle driving posture and ergonomics [22], the ROM is divided into three levels: comfortable range, less comfortable range, and uncomfortable range, and the specific angles are shown in Table 1. In different operating postures, the changes of joint angles of each of the human body parts are different. The division of comfort of joint angles is the basis of operating comfort evaluation as in Table 1.

### 2.3. GEP

#### 2.3.1. Brief Introduction of GEP

Refer to biological genetic gene expression patterns, Portuguese scientist Candida Ferreira proposed a revolutionary new member in the evolutionary computation family—GEP, which combined with the advantages of genetic algorithm and genetic programming [23, 24]. Because GEP is not dependent on the specific areas of problems, it has very strong robustness for kinds of problems, and it is widely applied to formula discovery, function mining, association rules discovery, factorization, sunspots forecasting, and other fields.

GEP is a kind of new data mining technique, which has ideal efficiency. Because GEP combines the advantages of genetic algorithm and genetic programming, its efficiency is higher than GA or GP 2~4 orders of magnitude in solving complex problems. Using adaptive random search method, GEP is able to discover formula which can describe data inherent law from the data, without relying on any prior knowledge, showing strong accuracy and universality. Research on improvement and application of GEP algorithm has been attracting more and more attention, but the GEP has not been applied in the field of operating comfort prediction. So, this paper attempts to carry out the research on operating comfort prediction model based on GEP.

In GEP, a computer program is coded into fixed length of linear symbol strings and then when calculating the individual fitness, chromosomes (genotype) are expressed as different shapes and sizes of expressing tree (phenotype). Gene is the basic unit to constitute chromosome. Chromosome represents the feasible solution to solve the problem and consists of one or more genes. Formalization definition of gene can be expressed as a six-tuple:

(2) $$\begin{array}{c} Gene=\left(\;\Omega ,F,T,\Phi ,h,f\;\right), \end{array}$$

where *Ω* represents the set of genetic elements, namely, linear string. *F* represents the function set and contains mathematical function and custom function and so forth. *T* represents the terminal set and generally includes variables, nonparameter functions, and constants. Φ represents the set of genetic operators, such as variation and translocation. *h* represents the length of gene head; the length of gene tail and total length of gene can be calculated by the length of gene head. *f* represents the fitness value, calculated by the fitness function.

#### 2.3.2. The Steps of GEP Algorithm

As one kind of evolutionary algorithm, the operation process of GEP is similar to genetic algorithm. First of all, randomly generate initial population containing a certain number of individuals, and evaluate the fitness of these chromosomes. Then, on the basis of the fitness valve, choose the individuals as the next generation of population, conduct genetic operation on the selected individuals, and generate offspring with new features. The new individuals enter into the next round of the survival of the iterative process, and the process is repeated until the terminal condition is satisfied. The main steps of function mining by GEP are as follows [25].

**Step 1 .**

Code the individuals and create the initial population. Population contains a number of individuals (chromosomes), and chromosome is composed of more than one gene (see Table 2). Genes are connected by linking function. Head contains function set (*F*) or terminal set (*T*), and tail contains terminal set (*T*).

**Step 2 .**

Calculate the fitness value. The fitness value of each individual is calculated by fitness function. And the fitness value reflects the extent of excellence of individual to achieve the optimal solution in the course of evolution. If the optimal individual meets ending condition, it should be transferred to the output and if not, it should be transferred to genetic operation steps, and then it produces offspring with new characteristics. In GEP, in order to evaluate the matching degree between the data calculated by the expression and training data, Ferreira put forward two evaluation models: the fitness function based on absolute error and the fitness function based on relative error.

**Step 3 .**

Carry out a series of genetic operations to produce a new generation of population using evolution principles to guide the evolution, including (1) keeping the best individual, (2) selection, (3) replication, (4) mutation, (5) transposition, and (6) recombination. If the mutation occurs in gene head, all the symbols can be selected. If the mutation happens in gene tail, only the terminal symbol can be selected. The snippets or string (transposable elements) combined by adjacent gene elements will be inserted into the other position of the chromosomes by transposition operator. Basic transposition operator contains IS, RIS, and gene transposition. Recombination is the process where two chromosomes will be selected from parent chromosomes randomly, interchange of some components, and generate new offspring. Basic recombination operator contains 1-point, 2-point, and gene recombination.

## 3. Case

Take the operating comfort evaluation of console layout of a certain type drilling rig as an example to illustrate the implementation of the proposed method. Use CATIA software to establish the human body and the product model, integrate the human physiological characteristics, simulate the operating postures, and realize the visualization of dynamic process in human-computer interaction. At the same time, making full use of ergonomic evaluation criteria and algorithm, operating comfort is analyzed and evaluated quantitatively. Using the data obtained from the simulation evaluation as training sample and validation sample, the relation model of joint angles and operating comfort is established based on GEP. Through limb angles to predict the comfort of operating posture, the basis of evaluation and optimization of human-machine interface layout design for cabin is provided.

### 3.1. Establish the Chinese Virtual Human Body Model in CATIA

The ergonomic design module of CATIA integrates four submodules: the human builder, human measurements editor, human activity analysis, and human posture analysis [26]. Human joints exist the maximum ROM, the software can test whether the location of manipulators within the reachable area; and quantify the comfortable degree. But CATIA only has five kinds of the human body model: the United States, Canada, France, Japan, and Korea. Therefore, in order to get accurate evaluation results, the Chinese virtual human body model needs to be established firstly.

Create a data file of the human body model which must follow certain form. A population file contains four segments; the form is as follows:

 MEAN_STDEV M (): this segment lists each part of the body size of the male.
 MEAN_STDEV F (): this segment lists each part of the body size of the female.
 CORR M (): this segment lists the correlative numerical values between each part of size variable of the male.
 CORR F (): this segment lists the correlative numerical values between each part of size variable of the female.

The segment of MEAN_STDEV needs each measurement numerical value of Chinese adult body size, including the mean and the standard deviation. Each item takes up one line with the pattern of “〈variable〉  〈mean〉  〈standard  deviation〉” to describe a variable.

The segment of CORR needs correlative numerical values between any pairs of variables; the correlation between any two variables is defined in −1.0~1.0. It expresses the dependencies between two variables. The absolute value of correlation is higher; the dependencies between variables are higher.

According to the above format, the human body dimension data from the Chinese standard GB10000-88 is wrote in order; a complete database file of the human body dimension can be established. Take  .sws as extension name, the file can be uploaded by user defined population database in CATIA. Detailed constructive process can be found in [27].

### 3.2. Operating Comfort Evaluation Based on CATIA

Imbedding the Chinese virtual human body model into human-machine interface layout scheme model, the human body model can be adjusted according to the selected percentile in real time. For different layout scheme, the human also has different operating posture. CATIA can adjust the virtual human body model to different operating posture fast and conveniently.

**Figure 4:**
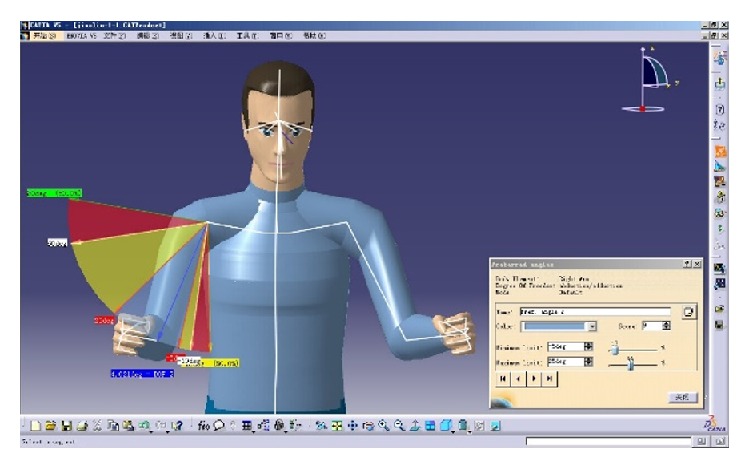
Set up the preferred angle of upper arm.

**Figure 5:**
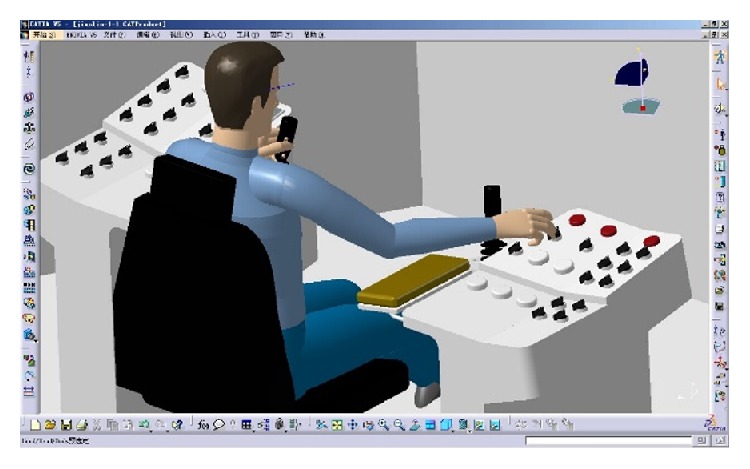
The simulation of operating posture.

**Figure 6:**
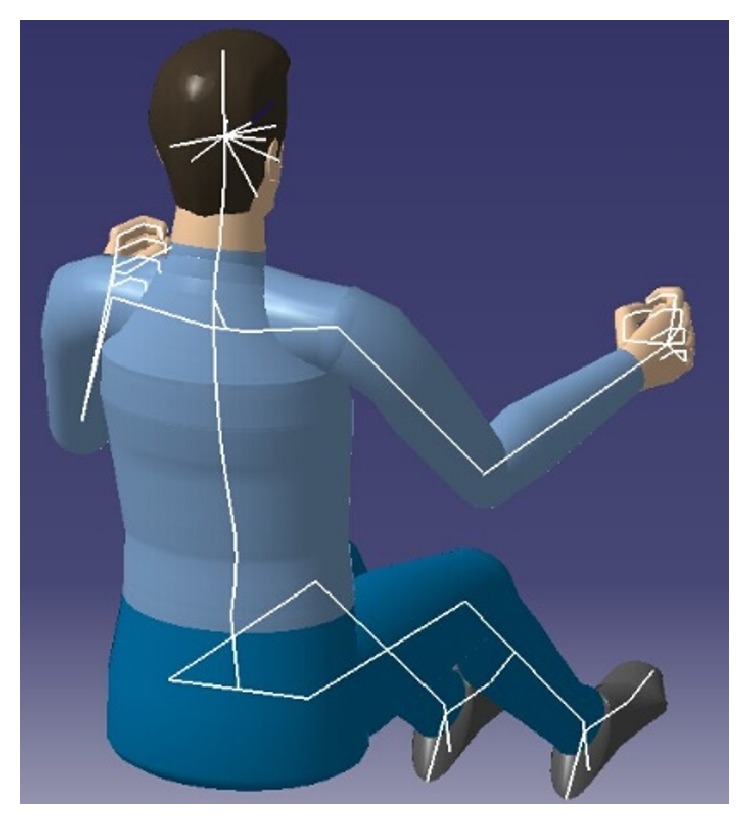
Certain operating posture.

**Figure 7:**
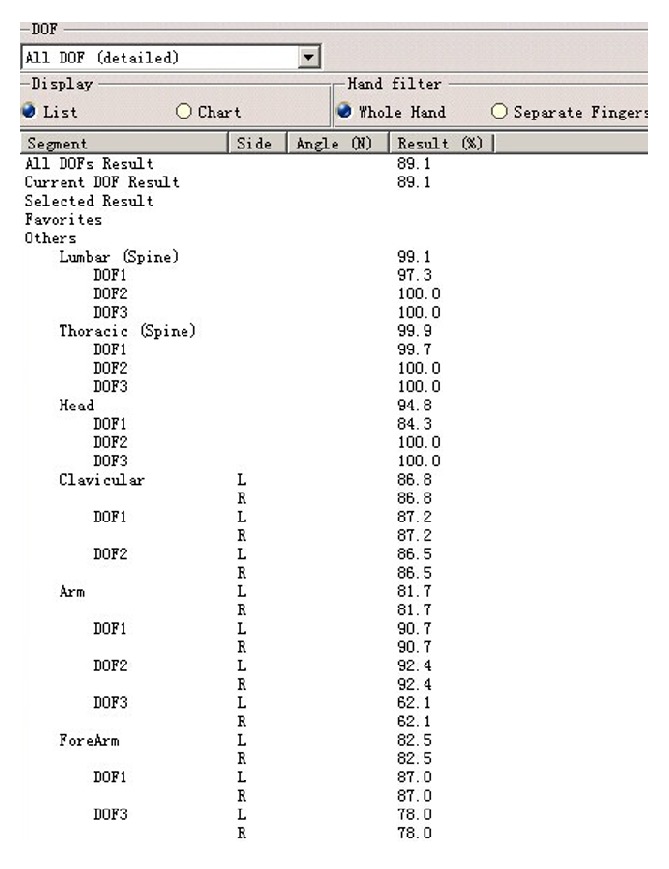
The corresponding comfort score of operating posture.

Before the posture evaluation, the preferred angle and corresponding score of each DOF of joints must be defined. When evaluating the comfort of body parts, based on the angle of DOF and score in current posture, the software will conclude evaluation score by interpolation and weighted arithmetic.

(*1) Set Up the Preferred Angle and Corresponding Score of Each DOF of Joints*. According to Table 1, to divide comfortable ROM of head, chest, waist, shoulder, upper arm, and forearm, edit the angle of locomotive parts of the human body, and set up the comfort score. For example, select the upper arm of the virtual human body model in Figure 4, edit the angular limitations and the preferred angles of DOF 2(adduction/abduction). The range is divided into three levels: comfortable range, less comfortable range, and uncomfortable range. The range is divided into five regions and different levels, respectively, show different colors. Meanwhile, in the blue area (−5°~25°), upper arm is comfortable and the score is 9 points, while, in the yellow area (−10°~−5°, 25°~60°), upper arm is less comfortable and the score is 7 points, whereas, in the red area (−18°~−10°, 60°~80°), upper arm is uncomfortable and the score is 5 points.

(*2) The Score of Operating Comfort*. Simulate the driller's operating process (see Figure 5) and evaluate the comfort in different operating posture. According to the operating task, edit the posture of head, torso, arm, leg, and foot. After posture editing (see Figure 6), the quantitative analysis of the virtual human model's posture is carried out.  Figure 7 shows the comfort score when the driller of 90 percentile handing control handles. In this operating posture, the comfort score of each part of the human body model is as follows: 9.48 (head), 9.99 (chest), 9.91 (waist), 8.68 (right shoulder), 8.17 (right upper arm), and 8.25 (right forearm); the overall comfort score is 8.91.

### 3.3. The Statistics and Analysis of Simulation Data

(*1) The Statistics of Comfort Score*. Take the right hand operating the console in Figure 5, for example; in turn, simulate the posture of operating the 22 manipulators in practical work. Take the average score of each DOF as comfort score of every part; the statistic results of comfort score are shown in Table 3.

(*2) Factor Analysis*. With 16 joint angles to describe the operating comfort being too complicated, dimension reduction is required. Factor analysis is a method of multivariate statistical analysis, which studies how to make numerous original variables condense into a few factors with the least amount of information loss and how to make the factors have certain named explanatory [28].

First of all, check whether the data is suitable for factor analysis. Input the data in Table 3 into SPSS Statistics 19.0 software; by Bartlett's test of sphericity and Kaiser-Meyer-Olkin (KMO) test, the relationship between variables is tested. The statistics observed value is 416.785 in Bartlett's test of sphericity; since the corresponding probability of *P* value is close to 0, less than the significance level of *α* (*α* equal to 0.05), it can be regarded as that there is significant difference between the correlation coefficient matrix and unit matrix. The value of KMO is 0.744; according to the KMO metrics provided by Kaiser, the original variables are appropriate to conduct factor analysis.

According to the principle of “usually select the number of eigenvalues as factor number when cumulative variance contribution rate is greater than 0.85,” the factors are extracted by the method of principal component analysis. Four factors are extracted; the corresponding cumulative variance contribution rate reaches 86.311% (shown in Table 4) and meets the above principle.

In Table 5, the data shown in bold in each column represent the joint angles as having high loading on the four factors, respectively. For example, *α*_12_, *α*_15_, and *α*_1_ have high load on the first factor; the first factor mainly explains these three variables of joint angle. In this way, the dimension of 16 variables is reduced to 4 comfort impact factors and can reflect most of the information of the original variables.

By regression method, the factor score coefficient is estimated. Component sore coefficient matrix is calculated, and the calculation formula of factor score is expressed as follows:

(3) x1=−0.189∗α1−0.094∗α2+⋯−0.323∗α15+0.321∗α16,x2=0.018∗α1−0.006∗α2+⋯+0.062∗α15−0.258∗α16,x3=0.037∗α1−0.126∗α2+⋯+0.003∗α15+0.006∗α16,x4=−0.036∗α1+0.331∗α2+⋯+0.189∗α15−0.366∗α16.

Factor analysis is used to reduce the variable (namely, joint angle) dimension and eliminate the correlation between the variables so as to reduce the independent variable inputting in GEP later. The data after dimension reduction are shown in Table 6.

### 3.4. Operating Comfort Prediction Model Based on GEP

Through the analysis of the data in Table 6, study the influence of the joint angles on operating comfort. Then, GEP algorithm is used for data mining, and the operating comfort prediction model of human-machine interface layout for cabin is established.

#### 3.4.1. Determine Function Set and Terminal Set

Determine the symbols representing chromosome; namely, choose function set and terminal set which suited the solution. The coding environment of GEP can be described as GEP = (*F*, *T*). Suppose function set *F* = {+, −, *∗*, /, Sqrt, Ln, *X*2, Avg2}, and common function includes arithmetic operators, elementary mathematics function, relational operators, and Boolean operator. Terminal set *T* = {*x*_1_, *x*_2_, *x*_3_,…, *x*_*n*_}, where *x*_1_, *x*_2_, *x*_3_,…, *x*_*n*_ represents the comfort impact factor set.

#### 3.4.2. Select Fitness Function

The evolution results in every generation are evaluated by fitness function; the individuals with bigger fitness value are retained and have a higher chance to reproduce. According to the characteristics of the problem and based on the mean square error (MSE), this paper constructed the fitness function. The smaller the MSE value the bigger the fitness value of individual. The largest fitness value is 1000. The fitness function is defined as follows:

(4) $$\begin{array}{c} f_{i}=\frac{1000}{E_{i}+1}, \end{array}$$

where *E*_*i*_ = (1/*m*)∑_*j*=1_^*m*^(*P*_*ij*_ − *T*_*j*_)^2^ is the MSE of experimental samples. *m* is the total number of training set samples; *P*_*ij*_ is the output value of the *j*th sample of the *i*th individual, calculated by the mathematical expressions acquired by GEP algorithm modeling; *T*_*j*_ is the target value of the *j*th sample.

The larger the fitness value of individual the better the individual. Stop condition of algorithm is the fact that the fitness value of best individual achieves the required accuracy or the program achieves the maximum evolutionary generations.

#### 3.4.3. Determine the Organizational Structure of Individual

Determine the genetic structure and the length of gene head. According to the complexity of the problem to define the length of gene head *h*, the length of gene tail *t* and the length of gene head *h* satisfy the relationship that *t* = *h* (*n* − 1) + 1. And *n* represents the maximum number of operation numbers in function set. So it can guarantee that the genes are legitimate. Suppose that a gene is composed of the elements in {+, −, *∗*, /, sin, cos, *x*_1_, *x*_2_, *x*_3_, *x*_4_}, so *n* = 2. If *h* = 6, then *t* = 6(2 − 1) + 1 = 7. Randomly generate a legitimate GEP gene as follows:

(5) $$\begin{array}{c} +\;+\;sin\;-\;*\;/\;x_{4}\;x_{3}\;x_{1}\;x_{2}\;x_{3}\;x_{2}\;x_{1}, \end{array}$$

where the top six represent the gene head, and the rest represent the gene tail.

**Figure 8:**
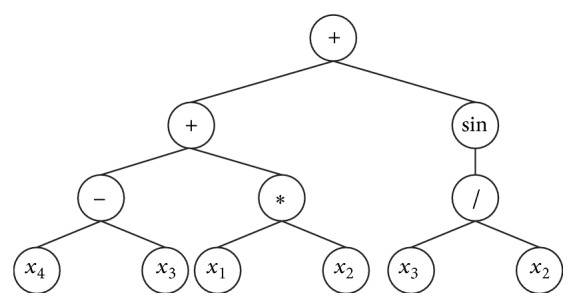
Gene expression tree.

The corresponding expression tree is shown in Figure 8.

In GEP coding, the length of each gene is fixed, including the front effective K-expression and the back of filler components. From top to bottom and left to right, the expression tree in Figure 8 is traversed, and K-expression of expression tree is obtained. The effective length of the gene has 12 characters: +  +  sin  −  *∗* / *x*_4_ *x*_3_ *x*_1_ *x*_2_ *x*_3_ *x*_2_. So the mathematical expression is as follows:

(6) $$\begin{array}{c} \left(\;x_{4}-x_{3}\;\right)+x_{1}*x_{2}+\sin\left(\;\frac{x_{3}}{x_{2}}\;\right). \end{array}$$

#### 3.4.4. Operating Comfort Prediction

**Figure 9:**
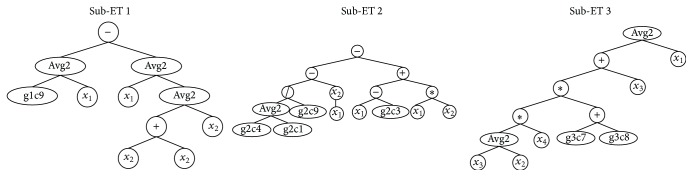
The sub-ET of optimal individual.

Determine the genetic control parameters before the algorithm running, including the size of population, the upper limit of evolution generation, and the probability of each genetic operator. The main operating parameters of GEP model are shown in Table 7. In function set, Sqrt represents square root; Ln represents return to natural logarithm of a number; *X*2 represents square; Avg2 represents mean of two variables. In terminal set, *x*_1_, *x*_2_, *x*_3_, and *x*_4_ represent four comfort impact factors extracted by factor analysis. 15 groups of simulation data are randomly selected from 22 groups of data in Table 6 as training set and the rest 7 groups as validation set. Through Visual Basic programming, the best individual is obtained after running multiple times, using computer with Inter Core i7-4500U CPU and 8 GB RAM. The best individual expression tree is shown in Figure 9; each expression tree represents a gene, and 3 genes are connected by linking function “+” to form a chromosome. The best individual translated into mathematical expression is as follows:

(7) fx=127.850+x1−12x1+12x2+x2+x2+1/21.406−5.9560.990−x12−x1−7.005+x1x2+1212x3+x2x4−2.590+3.303+x3+x1.

**Figure 10:**
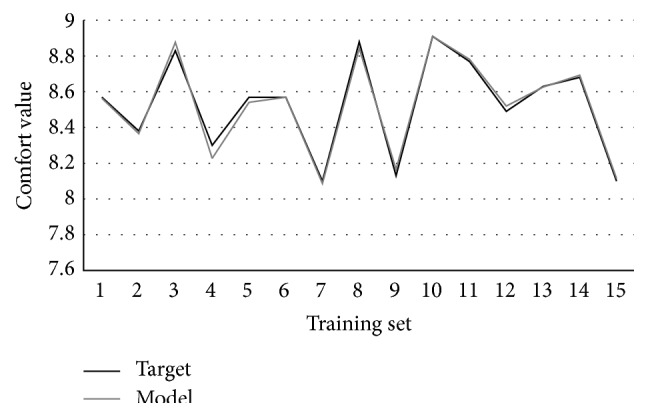
The fitting curve of training set.

Figure 10 shows the curve fitting of GEP algorithm in the training set. MSE and *R*-square are used to verify the validity of the algorithm and the ability of the prediction. The formulas are as follows:

(8) MSE=1n∑i=1nyi′−yi2,R-square=1−∑i=1nyi′−yi2∑i=1nyi−yavg2,

where *y*_*i*_′, *y*_*i*_, *y*_avg_ represent the predicted value, the actual value, and the actual average value, respectively.

**Figure 11:**
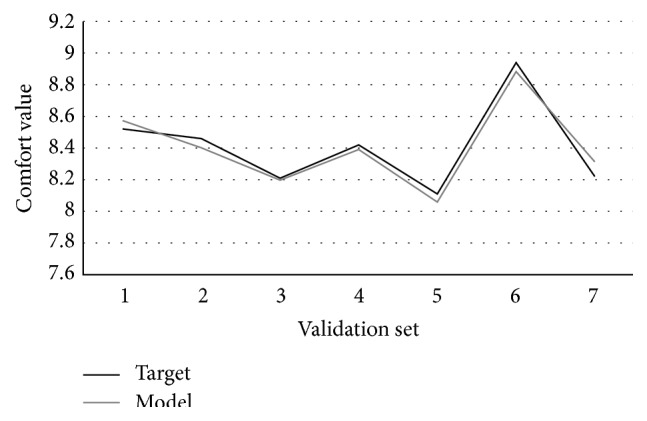
The fitting curve of validation set.

The range of *R*-square is [0, 1], the closer to 1, showing that the four variables have stronger ability to predict the operating comfort. The calculation result shows that the MSE and *R*-square of training set are 0.0009 and 0.9883, respectively. In order to verify the validity of GEP model, the rest 7 groups of data are taken into the model for verification. The curve fitting of validation set is shown in Figure 11. And the MSE and *R*-square are 0.0031 and 0.9538, respectively, which achieve the ideal effect.

### 3.5. Model Performance Comparison

**Figure 12:**
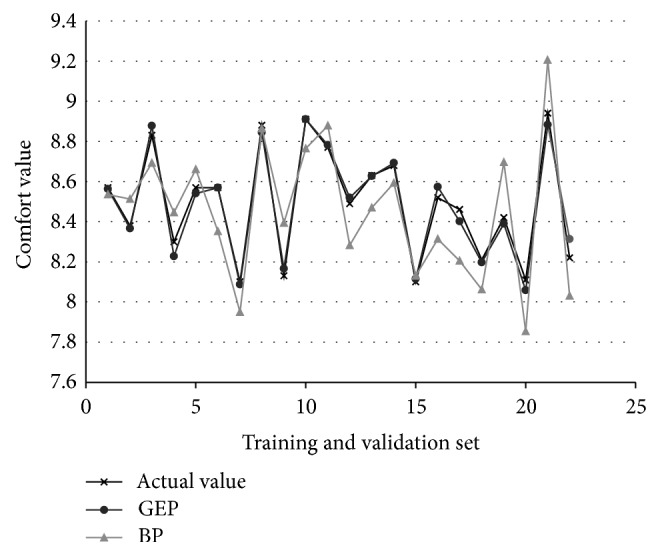
Comparison chart of actual value and predicted value by GEP and BP prediction model.

Back propagation neural network model and GEP algorithm model are used to predict the above 22 sets of data, respectively, and the predicted values and the relative error are shown in Table 8. The comparison chart of actual value and predicted value by two kinds of prediction model is shown in Figure 12. In Table 8, the average relative error of operating comfort prediction obtained by GEP model and BP model is 0.37% and 1.89%, respectively. In comparison, the average relative error of GEP model is smaller and the prediction accuracy is higher. So, the GEP model has high fitting degree.

### 3.6. Results and Discussion

**Figure 13:**
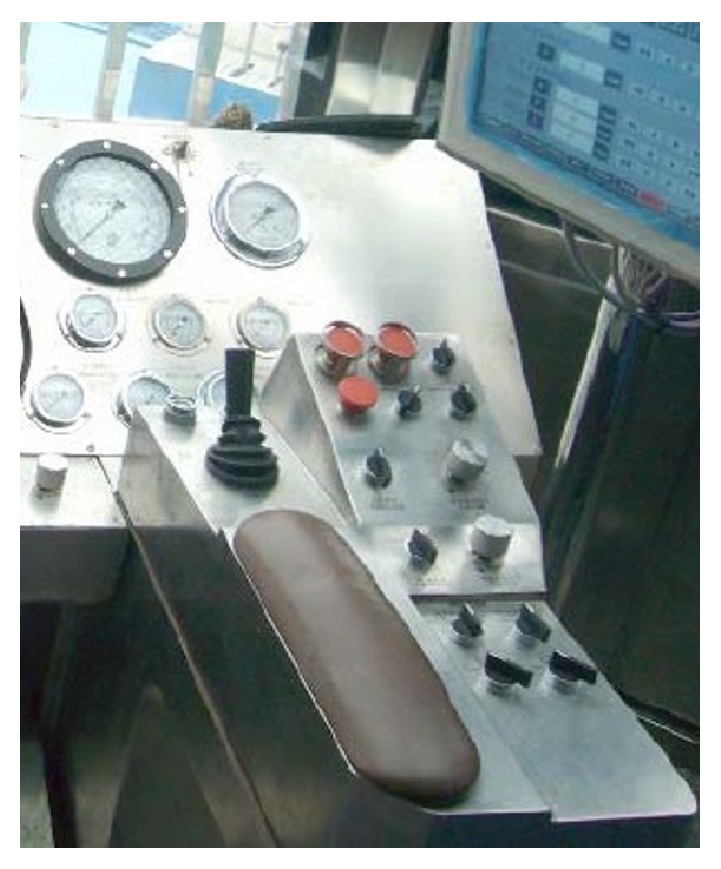
The case for verification.

In order to validate the presented operating comfort prediction method of human-machine interface layout, the method of questionnaire survey is adopted, and the operating comfort of operating 14 manipulators on console shown in Figure 13 is evaluated again. 20 drillers (all men) are invited to evaluate operating comfort with scoring criteria from 0 to 10. The questionnaire results are compared with the prediction results using the evaluation method proposed in this paper.

Table 9 shows that the deviation between the score of questionnaire and abovementioned method is among 0.04~0.51, and the average deviation of 14 operating comfort scores is 0.211. Therefore, the operating comfort prediction method of human-machine interface layout for cabin put forward in this paper can accurately predict the drillers' feel and assist the designer to design human-machine interface layout.

In addition, in accordance with the method of establishing the virtual human body model put forward in Section 3.1, other countries, regions, and even special population can be customized, which can be used for ergonomic evaluation of some particular products. The ROM can be divided according to different industries and job characteristics.

## 4. Conclusion

Comfort is a kind of subjective feeling, and it is difficult to quantify. In the process of operation, controlled by the feedback mechanism of the human body movement, the body always keeps each joint at a high comfort level as much as possible. Utilizing this adjustment mechanism, the operating comfort evaluation data is obtained by CATIA software. Then, GEP algorithm is applied for ergonomic analysis of human-machine interface layout for cabin. With 22 groups of evaluation data as the prediction model's data base, according to GEP algorithm to realize the complex functions' automatic modeling, the operating comfort prediction model is established. The example of operating comfort prediction of human-machine interface layout for driller control room proves that GEP has strong nonlinear and global search ability to find function, with the predicted results close to the target, and it has high prediction accuracy. With the limited training samples, GEP also can get accurate results. The comfort prediction model constructs the coupling relationship between joint angles and operating comfort, providing a solution for rapid assessment.

## Figures and Tables

**Table 1:** Divide the ROM of joints (°).

Parts of the body	Mode of activity	DOF	Comfortable range	Less comfortable range	Uncomfortable range
Head	Flexion (+)/extension (−)	DOF 1	0~12	−5~0, 12~20	−20~−5, 20~25
	Lateral left (+)/lateral right (−)	DOF 2	−5~5	−10~−5, 5~10	−20~−10, 10~20
	Rotation right (+)/rotation left (−)	DOF 3	−25~25	−35~−25, 25~35	−75~−35, 35~70

Chest	Flexion (+)/extension (−)	DOF 1	−10~10	10~15	—
	Lateral left (+)/lateral right (−)	DOF 2	−5~5	−10~−5, 5~10	−20~−10, 10~20
	Rotation right (+)/rotation left (−)	DOF 3	−10~10	−20~−10, 10~20	−70~−20, 20~70

Waist	Flexion (+)/extension (−)	DOF 1	−5~10	−10~−5, 10~20	20~40
	Lateral left (+)/lateral right (−)	DOF 2	−5~5	−10~−5, 5~10	—
	Rotation right (+)/rotation left (−)	DOF 3	−5~5	−10~−5, 5~10	—

Shoulder	Flexion (+)/extension (−)	DOF 1	−2~2	−5~−2, 2~5	−8~−5, 5~20
	Elevation (+)/depression (−)	DOF 2	−3~10	10~20	−8~−3, 20~53

Upper arm	Flexion (+)/extension (−)	DOF 1	−5~35	−20~−5, 35~90	−60~−20, 90~170
	Abduction (+)/adduction (−)	DOF 2	−5~25	−10~−5, 25~60	−18~−10, 60~80
	Medical rotation (+)/lateral rotation (−)	DOF 3	−5~5	−10~−5, 5~15	−20~−10, 15~97

Forearm	Flexion (+)/extension (−)	DOF 1	80~110	0~80, 110~115	115~140
	Pronation (+)/supination (−)	DOF 2	0~80	80~90	90~160

**Table 2:** The genetic structure of GEP.

Gene 1		Gene 2		⋯	Gene *n*	
Head	Tail	Head	Tail		Head	Tail
*F*/*T*	*T*	*F*/*T*	*T*	⋯	*F*/*T*	*T*

**Table 3:** The statistics of comfort score.

Limb	Head			Chest			Waist			Right shoulder		Right upper arm			Right forearm		Overall comfort score
DOF	DOF 1	DOF 2	DOF 3	DOF 1	DOF 2	DOF 3	DOF 1	DOF 2	DOF 3	DOF 1	DOF 2	DOF 1	DOF 2	DOF 3	DOF 1	DOF 2	
Angle (°)	*α*_1_	*α*_2_	*α*_3_	*α*_4_	*α*_5_	*α*_6_	*α*_7_	*α*_8_	*α*_9_	*α*_10_	*α*_11_	*α*_12_	*α*_13_	*α*_14_	*α*_15_	*α*_16_	
*m*_1_	0	0	0	0	0	0	4	0	0	−2	10	35	4	−13	63	84	8.91
*m*_2_	0	1	8	2	0	0	7	0	0	1	34	62	15	29	0	84	8.63
*m*_3_	−3	1	8	6	0	10	15	0	7	5	39	70	24	29	0	84	8.21
*m*_4_	0	2	9	10	0	12	22	0	10	−3	36	84	28	12	0	99	8.10
*m*_5_	5	1	6	2	0	0	2	0	0	1	6	50	28	12	32	99	8.94
*m*_6_	5	1	10	6	0	1	4	0	1	3	17	70	28	12	0	125	8.57
*m*_7_	8	1	10	4	0	0	3	0	0	1	17	34	24	12	49	124	8.83
*m*_8_	10	1	11	4	0	0	3	0	0	1	12	26	39	12	53	101	8.88
*m*_9_	10	1	14	4	0	0	3	0	0	4	10	22	50	12	47	101	8.77
*m*_10_	15	1	15	0	0	0	0	0	0	−1	5	−16	34	−20	88	108	8.68
*m*_11_	17	1	16	0	0	0	1	0	0	−2	2	−27	56	−20	81	108	8.49
*m*_12_	17	1	17	0	0	0	1	0	2	1	2	−16	54	−20	67	108	8.52
*m*_13_	0	2	11	4	−4	2	7	0	2	16	15	66	49	−6	0	135	8.22
*m*_14_	−1	2	9	4	−8	2	10	−2	2	13	25	68	57	−9	0	104	8.10
*m*_15_	−1	2	9	3	−8	2	10	−2	2	12	21	68	57	−9	5	105	8.13
*m*_16_	−1	2	11	4	−8	2	10	−4	2	12	20	68	56	−9	7	105	8.11
*m*_17_	1	2	11	4	−6	2	10	0	2	8	21	50	47	2	7	105	8.42
*m*_18_	1	2	11	6	−6	2	13	−2	2	11	19	50	61	2	7	100	8.30
*m*_19_	7	−1	13	6	0	2	13	0	5	3	19	22	39	−11	38	101	8.57
*m*_20_	8	−1	19	6	0	2	13	−1	6	2	19	42	39	−10	15	101	8.46
*m*_21_	11	−1	21	0	0	2	8	−1	6	−2	9	30	39	−11	21	122	8.57
*m*_22_	10	−1	23	1	0	2	8	−3	6	−3	19	42	39	−11	11	122	8.38

**Table 4:** Total variance explained.

Component	Initial eigenvalues			Extraction sums of squared loadings			Rotation sums of squared loadings		
	Total	% of variance	Cumulative %	Total	% of variance	Cumulative %	Total	% of variance	Cumulative %
1	6.716	41.975	41.975	6.716	41.975	41.975	3.974	24.835	24.835
2	3.498	21.864	63.839	3.498	21.864	63.839	3.851	24.069	48.904
3	2.496	15.597	79.437	2.496	15.597	79.437	3.351	20.947	69.851
4	1.100	6.875	86.311	1.100	6.875	86.311	2.634	16.460	86.311
5	0.865	5.405	91.716						
6	0.431	2.694	94.409						
⋮	⋮	⋮	⋮						
15	0.008	0.048	99.992						
16	0.001	0.008	100.000						

**Table 5:** Rotated component matrix.

	Component			
	1	2	3	4
*α*_12_	**0.901**	0.350	−0.127	0.145
*α*_15_	**−0.860**	−0.387	0.213	0.031
*α*_1_	**−0.792**	−0.358	0.270	−0.383
*α*_7_	0.357	**0.900**	−0.166	0.013
*α*_9_	0.113	**0.886**	0.070	−0.396
*α*_6_	0.221	**0.881**	0.095	0.125
*α*_11_	0.621	**0.652**	0.091	0.238
*α*_4_	0.422	**0.631**	0.040	0.292
*α*_13_	−0.330	−0.077	**−0.895**	−0.014
*α*_5_	−0.319	0.008	**0.893**	−0.241
*α*_10_	0.432	−0.095	**−0.750**	0.323
*α*_8_	−0.240	−0.113	**0.692**	0.357
*α*_14_	0.560	0.119	**0.564**	0.485
*α*_3_	−0.458	0.014	−0.040	**−0.842**
*α*_2_	0.210	−0.093	−0.423	**0.752**
*α*_16_	0.143	−0.453	−0.166	**−0.593**

**Table 6:** The data about the joint angles after dimension reduction.

	Factor 1	Factor 2	Factor 3	Factor 4	Overall comfort score
*m*_1_	−0.168	0.062	0.456	0.337	8.91
*m*_2_	0.301	0.068	0.532	0.391	8.63
*m*_3_	0.142	0.699	0.401	0.406	8.21
*m*_4_	0.035	0.957	0.322	0.283	8.10
*m*_5_	0.041	−0.084	0.380	0.338	8.94
*m*_6_	0.423	−0.101	0.365	−0.019	8.57
*m*_7_	0.122	−0.128	0.409	0.110	8.83
*m*_8_	−0.196	0.047	0.316	0.332	8.88
*m*_9_	−0.231	0.061	0.212	0.295	8.77
*m*_10_	−0.581	0.020	0.209	0.188	8.68
*m*_11_	−0.720	0.082	0.087	0.192	8.49
*m*_12_	−0.627	0.109	0.064	0.107	8.52
*m*_13_	0.379	−0.090	−0.168	0.040	8.22
*m*_14_	0.173	0.193	−0.421	0.279	8.10
*m*_15_	0.149	0.166	−0.416	0.265	8.13
*m*_16_	0.191	0.186	−0.515	0.134	8.11
*m*_17_	0.067	0.185	−0.112	0.344	8.42
*m*_18_	0.018	0.296	−0.331	0.332	8.30
*m*_19_	−0.232	0.465	0.230	−0.036	8.57
*m*_20_	−0.086	0.487	0.186	−0.292	8.46
*m*_21_	−0.036	0.206	0.224	−0.571	8.57
*m*_22_	0.118	0.246	0.145	−0.737	8.38

**Table 7:** GEP parameter set.

Parameter names	Parameter values
Function set	*F* = {+, −, *∗*, /, Sqrt, Ln, *X*2, Avg2}
Terminal set	*T* = {*x*_1_, *x*_2_, *x*_3_, *x*_4_}
Generation	1000
Number of individuals	30
Number of genes	3
Linking function	+
Head size	8
Mutation rate	0.002
One-point/two-point/gene recombination rate	0.003
IS/RIS/gene transposition rate	0.005
Numerical constant	(−10, 10)

**Table 8:** Comparison of predicted results by GEP and BP.

Number	Sample	Factor 1	Factor 2	Factor 3	Factor 4	Overall comfort score			Relative error	
						Actual value	Predicted value		GEP	BP
							GEP	BP		
1	*m*_6_	0.423	−0.101	0.365	−0.019	8.57	8.5630	8.5353	−0.0008	−0.0040
2	*m*_22_	0.118	0.246	0.145	−0.737	8.38	8.3658	8.5124	−0.0017	0.0158
3	*m*_7_	0.122	−0.128	0.409	0.110	8.83	8.8769	8.6929	0.0053	−0.0155
4	*m*_18_	0.018	0.296	−0.331	0.332	8.30	8.2269	8.4456	−0.0088	0.0175
5	*m*_21_	−0.036	0.206	0.224	−0.571	8.57	8.5408	8.6613	−0.0034	0.0107
6	*m*_19_	−0.232	0.465	0.230	−0.036	8.57	8.5690	8.3524	−0.0001	−0.0254
7	*m*_14_	0.173	0.193	−0.421	0.279	8.10	8.0867	7.9501	−0.0016	−0.0185
8	*m*_8_	−0.196	0.047	0.316	0.332	8.88	8.8441	8.8638	−0.0040	−0.0018
9	*m*_15_	0.149	0.166	−0.416	0.265	8.13	8.1654	8.3935	0.0044	0.0324
10	*m*_1_	−0.168	0.062	0.456	0.337	8.91	8.9099	8.7646	0.0000	−0.0163
11	*m*_9_	−0.231	0.061	0.212	0.295	8.77	8.7820	8.8782	0.0014	0.0123
12	*m*_11_	−0.720	0.082	0.087	0.192	8.49	8.5195	8.2823	0.0035	−0.0245
13	*m*_2_	0.301	0.068	0.532	0.391	8.63	8.6264	8.4704	−0.0004	−0.0185
14	*m*_10_	−0.581	0.020	0.209	0.188	8.68	8.6929	8.5924	0.0015	−0.0101
15	*m*_4_	0.035	0.957	0.322	0.283	8.10	8.1147	8.1305	0.0018	0.0038
16	*m*_12_	−0.627	0.109	0.064	0.107	8.52	8.5734	8.3132	0.0063	−0.0243
17	*m*_20_	−0.086	0.487	0.186	−0.292	8.46	8.4013	8.2050	−0.0069	−0.0301
18	*m*_3_	0.142	0.699	0.401	0.406	8.21	8.1966	8.0616	−0.0016	−0.0181
19	*m*_17_	0.067	0.185	−0.112	0.344	8.42	8.3905	8.6967	−0.0035	0.0329
20	*m*_16_	0.191	0.186	−0.515	0.134	8.11	8.0588	7.8540	−0.0063	−0.0316
21	*m*_5_	0.041	−0.084	0.380	0.338	8.94	8.8832	9.2055	−0.0064	0.0297
22	*m*_13_	0.379	−0.090	−0.168	0.040	8.22	8.3137	8.0311	0.0114	−0.0230

**Table 9:** The comparison of evaluation results.

Manipulator number	The name of the manipulator	The score of questionnaire	The score of this method	Deviation
1	Gen E-Stop	8.50	8.42	0.08
2	VFD E-Stop	8.00	8.21	0.21
3	E-Brake	9.00	8.95	0.05
4	Parking Brake	8.50	8.87	0.37
5	Left Cathead	8.00	8.04	0.04
6	Right Cathead	8.00	8.11	0.11
7	RT Inertia Brake	9.00	8.84	0.16
8	RT Motor Control	8.50	8.76	0.26
9	RT Speed Setting	8.50	8.81	0.31
10	RT Torque Limit	9.00	8.68	0.32
11	Rotary Cathead	8.50	8.33	0.17
12	Air Spinner	8.00	8.21	0.21
13	Spare 1	8.00	8.15	0.15
14	Spare 2	7.50	8.01	0.51
